# A Simulation Study of Triband Low SAR Wearable Antenna

**DOI:** 10.3390/mi14040819

**Published:** 2023-04-05

**Authors:** Wazie M. Abdulkawi, Asad Masood, N. Nizam-Uddin, Mohammad Alnakhli

**Affiliations:** 1 Department of Electrical Engineering, College of Engineering in Wadi Addawasir, Prince Sattam Bin Abdulaziz University, Wadi Addawasir 11991, Saudi Arabia; 2 Electrical Engineering Department, HITEC University, Taxila 47080, Punjab, Pakistan; 3 Biomedical Engineering Department, HITEC University, Taxila 47080, Punjab, Pakistan

**Keywords:** industrial, scientific, and medical (ISM) band, X-band, microstrip antenna, wireless communication, wearable, specific absorption rate (SAR)

## Abstract

The proposed paper presents a flexible antenna that is capable of operating in several frequency bands, namely 2.45 GHz, 5.8 GHz, and 8 GHz. The first two frequency bands are frequently utilized in industrial, scientific, and medical (ISM) as well as wireless local area network (WLAN) applications, whereas the third frequency band is associated with X-band applications. The antenna, with dimensions of 52 mm × 40 mm (0.79 λ × 0.61 λ), was designed using a 1.8 mm thick flexible kapton polyimide substrate with a permittivity of 3.5. Using CST Studio Suite, full-wave electromagnetic simulations were conducted, and the proposed design achieved a reflection coefficient below −10 dB for the intended frequency bands. Additionally, the proposed antenna achieves an efficiency value of up to 83% and appropriate values of gain in the desired frequency bands. In order to quantify the specific absorption rate (SAR), simulations were conducted by mounting the proposed antenna on a three-layered phantom. The SAR_1g_ values recorded for the frequency bands of 2.45 GHz, 5.8 GHz, and 8 GHz were 0.34, 1.45, and 1.57 W/Kg respectively. These SAR values were observed to be significantly lower than the 1.6 W/Kg threshold set by the Federal Communication Commission (FCC). Moreover, the performance of the antenna was evaluated by simulating various deformation tests.

## 1. Introduction

Wearable antennas are gaining popularity in body-centric communication (BCC). The BCC defines three main realms of communication: on-body, off-body, and in-body communication, based on how wireless devices are connected. This categorization aligns with the IEEE 802.15 standardization group [1]. In-body communication refers to communication between external devices and medical implants, while on-body communication pertains to communication between body-worn devices. Body-worn antennas operate in close proximity to the human body, which affects their performance characteristics such as reflection coefficient, gain, efficiency, directivity, radiation pattern, and specific absorption rate (SAR) [2].

The choice of materials is a crucial factor in designing wearable antennas. Ideally, wearable antennas should be lightweight, low profile, compact, user friendly, and low maintenance. They should also be able to withstand mechanical stresses and deformations. Various materials have been used as antenna substrates due to their favorable electrical, chemical, and mechanical properties for the human body, including textiles [3], silk [4], nylon [5], leather [6], wash cotton [7], denim [8], polymer, fleece, and paper [9]. In our study, we utilized kapton polyimide with a relative permittivity of 3.5 as the substrate material for the proposed antenna.

Due to the interaction of a wearable antenna with the human body, the gain, efficiency, bandwidth, and driving impedance of the wearable antenna is notably affected. Additionally, a part of the electromagnetic radiation from the antenna penetrates the skin layer and interacts with tissue yielding in heating effect. This is quantified as SAR measured in W/Kg. A low value of SAR is desirable to have minimum side effects as defined by FCC. Various approaches have been adopted by researchers to minimize SAR. Authors in [10,11,12] used a modified ground plane to minimize SAR. Authors in [13,14,15] adopted metamaterials, electromagnetic bandgap (EBG) structures in [16,17,18], artificial magnetic conductors (AMC) in [19,20], and a high-impedance surface (HIS) approach in [21] to reduce side and back lobe radiations, thereby reducing the SAR value significantly. A maximum SAR value of 2 W/Kg is recommended when 10 g of mass is considered, while a maximum SAR value of 1.6 W/Kg for 1 g of tissue.

The advancement of wireless technology has enabled numerous systems to function across multiple frequency bands. Examples of such systems include satellite navigation, wireless LANs, and ultrawideband (UWB) systems [22]. Therefore, a single UWB or multiband antenna that possesses high gain, efficiency, and consistent radiation characteristics, along with an adequate impedance bandwidth, is worth considering as it can support multiple wireless communication systems [23]. However, compared to UWB antennas, it is more challenging to design and implement multiband antennas since they require precise control of the impedance bandwidth within the desired frequency bands. This is done to prevent unnecessary exposure of the wide bandwidth, which is a notable feature of UWB antennas [24,25].

This study, therefore, proposes a multiband wearable antenna that uses flexible kapton polyimide material as a substrate. The antenna is capable of operating in wireless body area networks (WBAN), WLAN, and X-bands. To investigate the specific absorption rate (SAR), we used a three-layer phantom composed of skin, fat, and muscle. Our results show that the achieved SAR values are below the threshold set by the FCC [26].

This paper is organized into several sections. Section 2 presents the proposed antenna design. A parametric study is detailed in Section 3. In Section 4, the results of the antenna’s gain, efficiency, surface current distribution, and bandwidth are discussed. Section 5 covers various tests conducted on the antenna, including wet, undercover, compression, and bending tests. The section also includes discussions on SAR analysis and transmission losses. Section 6 offers a comparison of the proposed design with other related works. Finally, Section 7 provides the conclusion.

## 2. Design Methodology

During the past three decades, planar antennas, specifically microstrip antennas, have gained widespread usage. Despite having a narrow bandwidth, researchers have directed their attention towards microstrip patch antennas due to their distinct benefits, such as low manufacturing costs, light weight, and ease of production.

The miniaturization of microstrip antennas has emerged as a significant area of interest among antenna designers in recent decades. Typically, the length of a conventional antenna that operates at a given frequency is on the order of half the wavelength of that frequency. However, this length is practically not suitable for several applications such as radio frequency identification (RFID), and the internet of things (IoT) [27].

The miniaturization of antennas with multiband capabilities is an active research area, particularly for wireless devices that require compact and efficient antenna systems. There are several techniques that researchers are exploring to achieve this goal, such as using metamaterials, reconfigurable antennas, and fractal geometries [28,29]. Additionally, the introduction of specialized antennas such as fork antennas can also achieve miniaturization [19,30].

**Figure 1:**
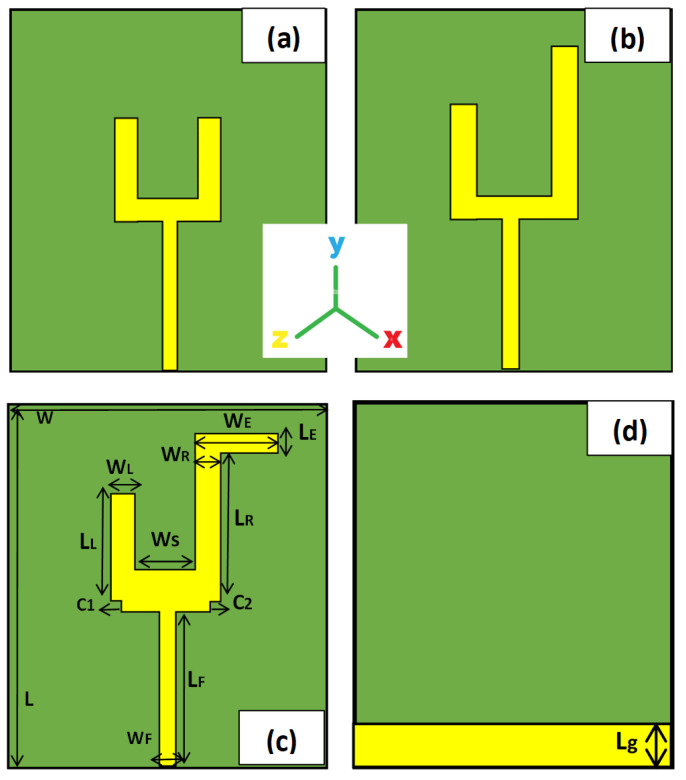
Antenna design evolution: (**a**) Symmetric U-shape, (**b**) A-symmetric U-shape, (**c**) Proposed antenna, and (**d**) Ground plane.

**Figure 2:**
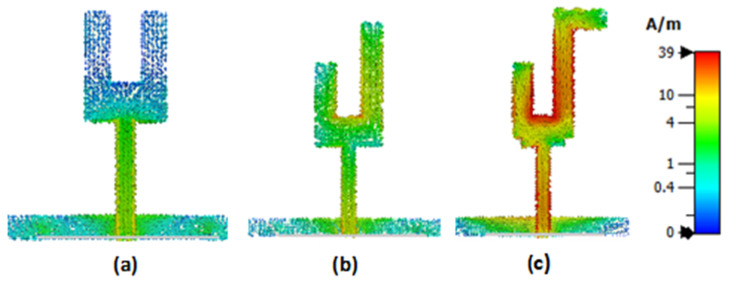
Distribution of surface current during antenna design evolution: (**a**) Initial design, (**b**) Modified design, and (**c**) Proposed design.

**Figure 3:**
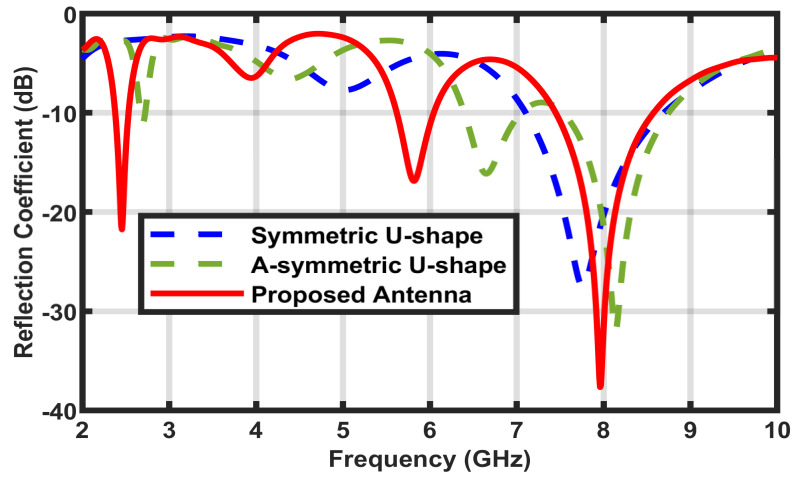
The return loss of the proposed antenna at various design steps.

The antenna design presented in this study is derived from a typical fork antenna design. The overall design evolution is presented in Figure 1. As an initial design, a symmetric U-shaped fork antenna fed at the centre was adopted, resulting in a frequency resonance at 7.8 GHz. At this stage, the surface current distribution was analyzed, and it was observed from Figure 2a that the radiating arms had low current intensity. To improve the concentration of current intensity, the antenna’s symmetry along the y-axis was modified, resulting in an enhanced current distribution. The current intensity was further increased by incorporating a horizontal stub on the right arm of the antenna, as shown in Figure 2c. The shaping of the current distribution was carried out carefully to attain resonating bands at three desired frequencies, namely 2.45 GHz, 5.8 GHz, and 8.0 GHz, as depicted in the return loss graph in Figure 3.

Table 1 presents the optimized dimensions of stubs, cuts, and the ground plane for the triband functioning of the antenna. L_L_, L_R_, L_E_, and L_F_ are the lengths of the stubs, horizontal strip, and feed line, respectively, while W_L_, W_R_, W_E_, and W_F_ are their corresponding widths. W_S_ is the spacing between the stubs. L_g_ represents the length of the ground plane.

## 3. Parameterized Study

**Figure 4:**
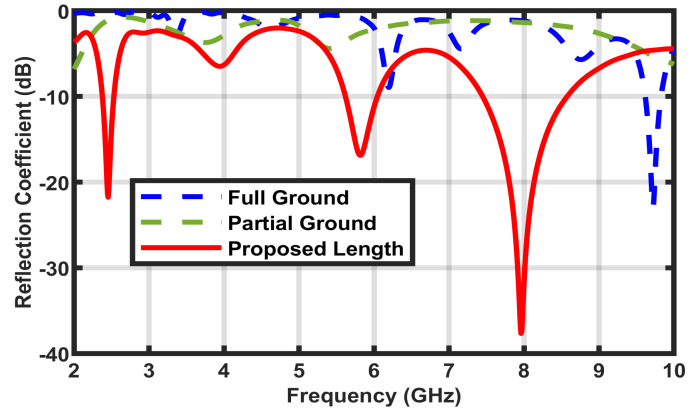
Effect of varying the length of the ground plane.

By changing various parameters of the antenna design, some useful insights can be comprehended. The return loss of the antenna is investigated by changing the length of the ground plane, as depicted in Figure 4. The transition from single to multi-resonance occurs by varying the length of the ground plane. A full ground enables the antenna to operate at a single resonance frequency of 9.8 GHz. The middle resonance frequency starts appearing when the antenna is operated for partial ground. When the length of the ground plane is further reduced to a value of 3.2 mm, the antenna exhibits a third resonance frequency in the ISM band.

**Figure 5:**
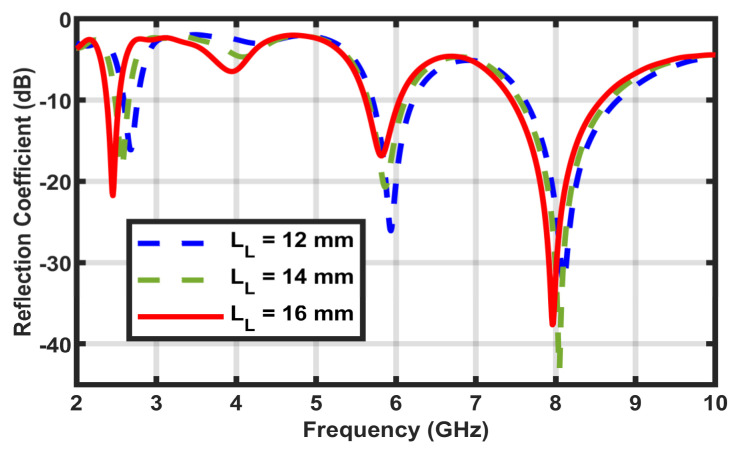
Effect of varying the length of left stub.

**Figure 6:**
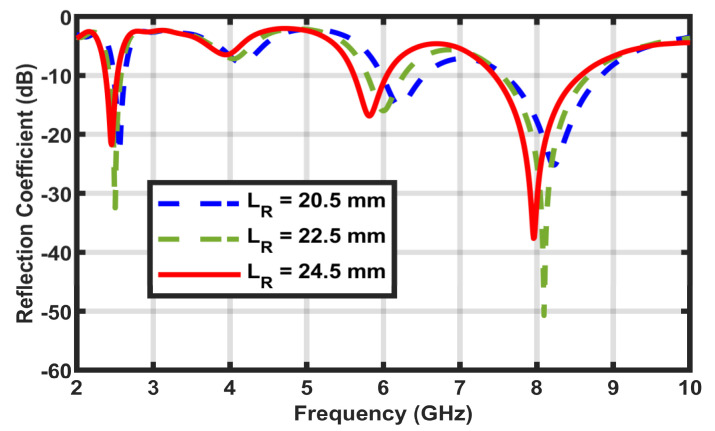
Effect of varying the length of the right stub.

The length of the stubs plays a crucial role in the frequency response of the antenna. Modifying the length of the stubs causes a shift in the center of resonating frequencies, providing frequency-tuning capabilities for the proposed antenna. As depicted in Figure 5, adjusting the length of the left stub for the ISM band alters the center frequency. On the other hand, Figure 6 demonstrates that modifying the length of the right stub leads to the modification of the center frequencies of the second ISM and X-bands.

**Figure 7:**
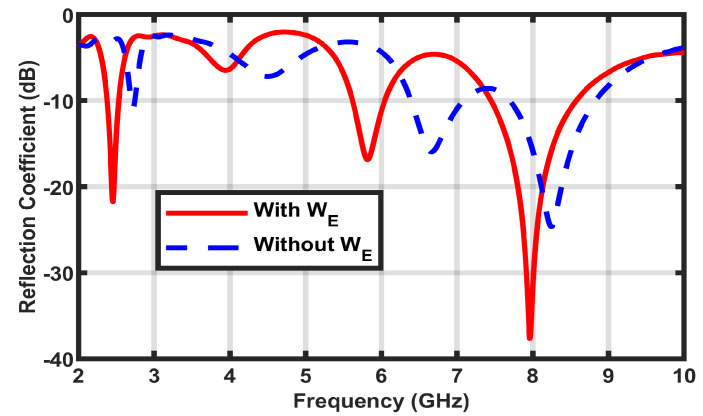
Effect of adding a horizontal strip on the reflection coefficient.

The addition of a horizontal stub to the radiating patch results in the modification of the return loss characteristics of the antenna. This stub was introduced to provide band-pass filtering characteristics to the antenna radiation by creating specific current paths that facilitate efficient radiation in the desired frequency bands. This effect is demonstrated in Figure 7.

**Figure 8:**
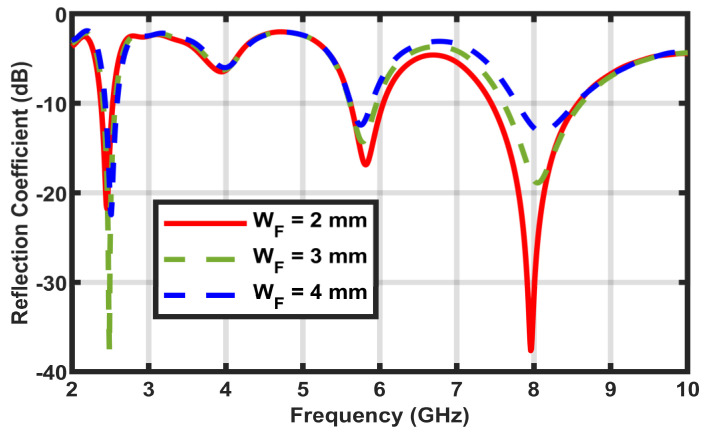
Effect of varying the width of the feed line on the reflection coefficient.

The dimensions of the feed line are a crucial consideration in antenna design as they impact impedance matching. In our design, a wider feed-line width leads to a lower matching impedance, while a narrower one results in a higher matching impedance. The feed-line width of 2 mm is found to be optimal, as shown in Figure 8.

## 4. Other Results

This section presents the simulation results of various performance parameters of the proposed antenna.

### 4.1. Comparison of Antenna Performance in the HFSS Computational Tool

**Figure 9:**
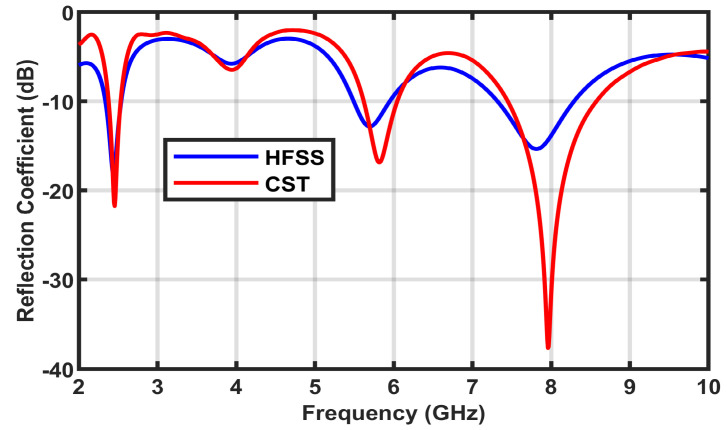
Comparison of the antenna in HFSS and CST.

In order to validate the functionality of the proposed design, the antenna was designed and simulated using the HFSS computational package, which is based on the finite element method (FEM). The comparison of the results is shown in Figure 9. The achieved results are in close agreement with each other, although HFSS exhibits a slight decrease in impedance matching. This is primarily due to variations in the dimensions of the radiation box available in both tools.

### 4.2. Efficiency and Gain of the Proposed Antenna

**Figure 10:**
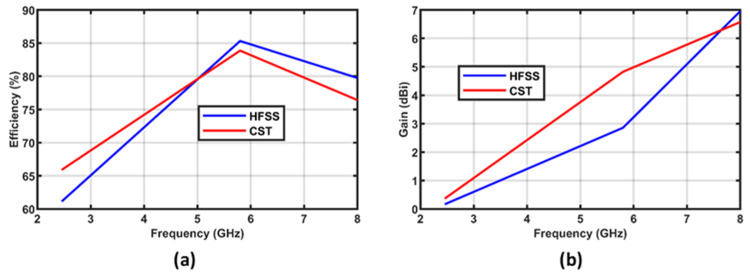
The simulated (**a**) efficiency and (**b**) gain of the proposed antenna.

The efficiency and gain of the proposed antenna were computed using both CST and HFSS simulation software. The results, as illustrated in Figure 10, show a slight variation in gain when computed in HFSS. The efficiency for 2.45 GHz is 65.8%, while for 5.8 GHz and 8.0 GHz, it is 83.8% and 76.4%, respectively. Similarly, the gains for these resonating frequencies are 0.36 dBi, 4.82 dBi, and 6.57 dBi, respectively.

### 4.3. Simulation of Front-to-Back Ratio (FBR)

**Figure 11:**
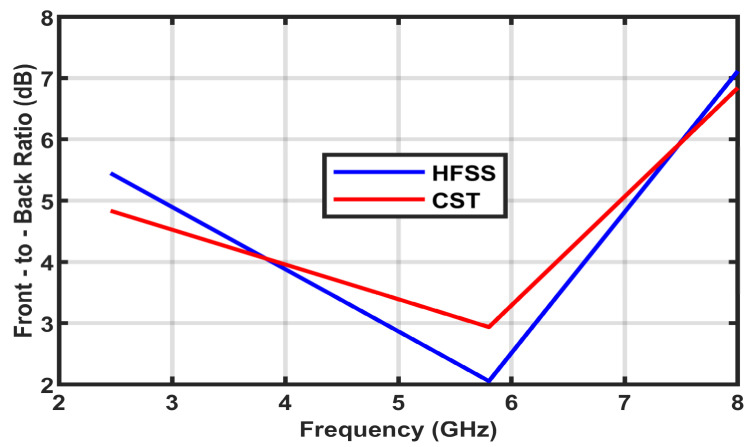
Analysis of antenna’s front-to-back ratio (FBR) in HFSS and CST.

FBR is a measure of the directional performance of an antenna. It describes the ratio of the power radiated in the forward direction compared to the power radiated in the opposite, or backward, direction. A higher FBR ratio indicates that the antenna radiates more power in the desired direction and less power in the opposite direction, which is often desirable in antenna applications. The FBR characteristics of the antenna were simulated using both CST and HFSS, as shown in Figure 11. Compared to 5.8 GHz, the FBR values were relatively greater for 2.45 GHz and 8.0 GHz.

### 4.4. Surface Current Distribution and 2D Radiation Pattern

**Figure 12:**
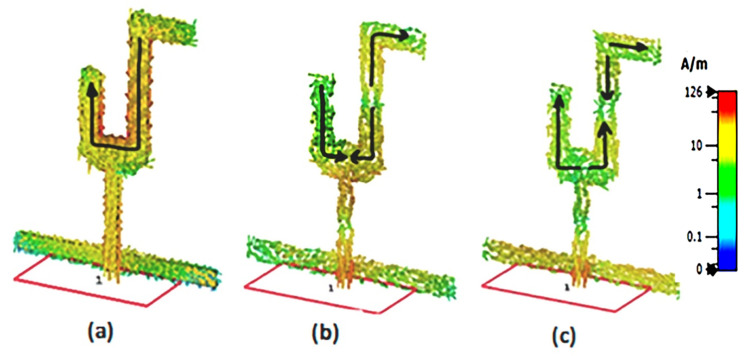
Surface current distribution at resonating frequencies of (**a**) 2.45 GHz, (**b**) 5.8 GHz, (**c**) 8.0 GHz.

Figure 12 presents the surface current distribution of the proposed antenna. At a frequency of 2.45 GHz, the antenna exhibits an intensified current distribution at the center of both stubs, as depicted in Figure 12a, where the current flows uni-directionally. In Figure 12b, the currents in the two arms oppose each other, and due to the uneven arm length, an additional current is generated in the horizontal stub, resulting in the generation of a second resonance frequency at 5.8 GHz. Finally, Figure 12c shows that the current distribution follows three distinct paths, leading to the generation of three resonant frequencies.

**Figure 13:**
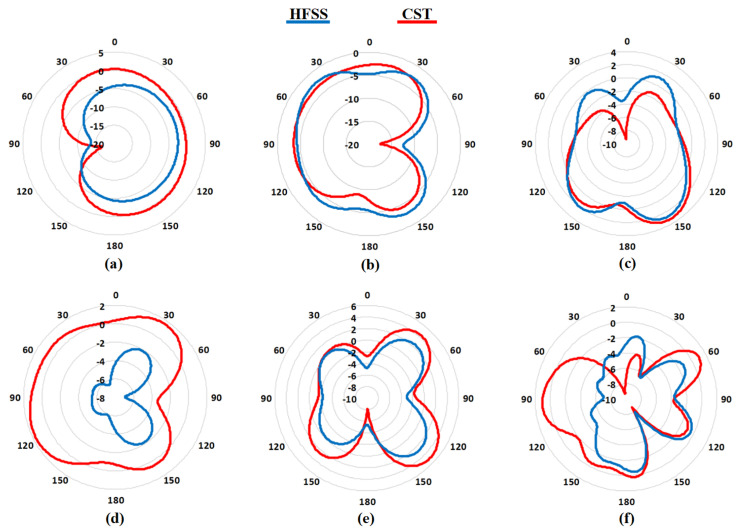
Simulation of antenna’s radiation pattern at frequencies of 2.45 GHz, 5.8 GHz, and 8.0 GHz: (**a**–**c**) E-Plane and (**d**–**f**) H-Plane.

Figure 13 illustrates the 2D radiation pattern of the proposed antenna in free space for three resonant frequencies, namely 2.45 GHz, 5.8 GHz, and 8.0 GHz (from left to right in Figure 13), and in two principal planes, namely E-plane and H-plane using CST and HFSS. The E-plane results are shown in Figure 13a–c and that of the H-plane in Figure 13d–f. It is apparent from the figure that the antenna exhibits nearly omnidirectional radiation in both planes at the lower frequency of 2.45 GHz, primarily due to the unidirectional current flowing in the antenna at this frequency. However, for the two higher frequencies (5.8 GHz and 8.0 GHz), the antenna departs from its omnidirectional radiation characteristics, owing to the multidirectional distribution of current in the antenna’s arms.

### 4.5. Bandwidth Analysis

**Figure 14:**
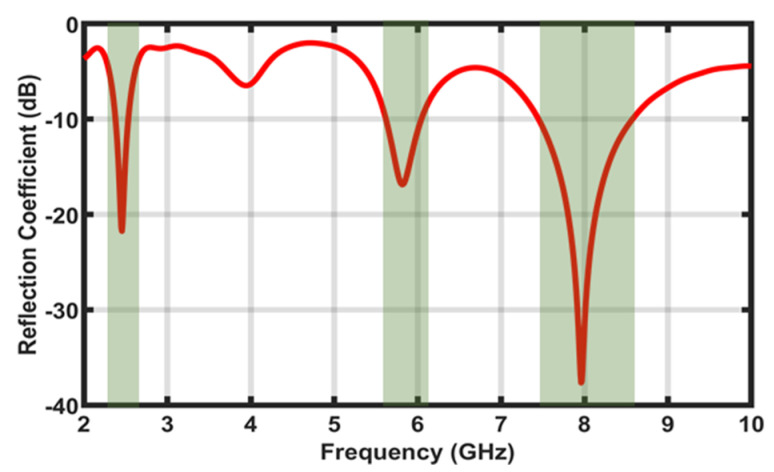
Impedance bandwidth of the proposed antenna.

In this section, we will discuss the impedance bandwidth offered by the antenna at its resonance bands. Figure 14 shows that the antenna has a relatively compact impedance bandwidth of 6.73% (2.37–2.54 GHz) in the first resonance band, which is narrower than the bands centered at 5.8 GHz and 8.0 GHz. However, at 8.0 GHz, the antenna exhibits a wider bandwidth of 13.81% (7.45–8.56 GHz). Table 2 presents the bandwidth values of the antenna at the three center frequencies, along with the S_11_ parameter, which indicates the level of impedance matching that the antenna can achieve. In our case, the antenna demonstrates high impedance matching at 8.0 GHz.

## 5. Testing

A body-worn antenna may undergo different deformations and distortions in real-life scenarios which can affect its performance. Here we present some cases.

### 5.1. Compression Test

**Figure 15:**
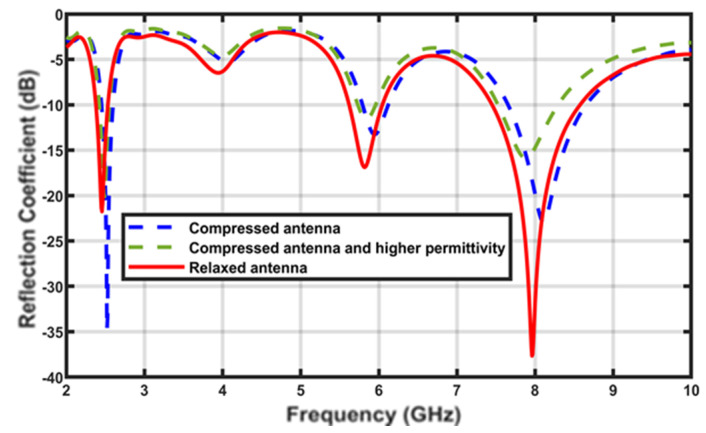
Compression analysis of the antenna by varying the thickness and permittivity.

When an external force is applied to an antenna, it may experience compression, primarily affecting its substrate. To investigate the effects of substrate thickness on antenna properties, we reduced it from 1.8 mm to 1.35 mm and observed a shift in the return loss of the antenna towards the right, as demonstrated in Figure 15. However, compression can enhance the permittivity of the substrate. To leverage this, we increased the substrate’s permittivity by 20%, from 3.5 to 4.25. As shown in Figure 15, this adjustment restored the return loss to its original values.

### 5.2. Substrate Variability Test

**Figure 16:**
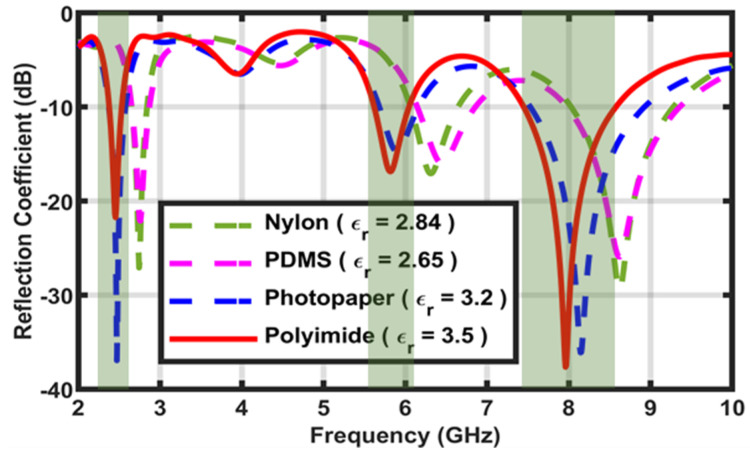
The return loss of the antenna for different substrate materials.

**Figure 17:**
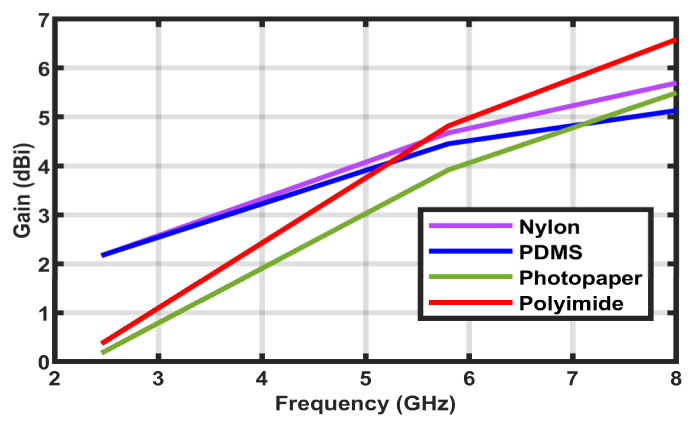
Antenna gain for various substrate materials.

To assess the versatility of the proposed antenna design, we evaluated its performance with substrates other than kapton polyimide. In this experiment, we replaced the substrate with materials of the same thickness, such as nylon, PDMS, and photopaper. Interestingly, we observed that the return loss characteristics deviated to a lesser extent, with resonances almost aligning with the target frequency bands, as illustrated in Figure 16. Additionally, Figure 17 displays the antenna gains for these substrate materials, revealing that Nylon exhibits a relatively higher gain than the others.

### 5.3. Bending Test

**Figure 18:**
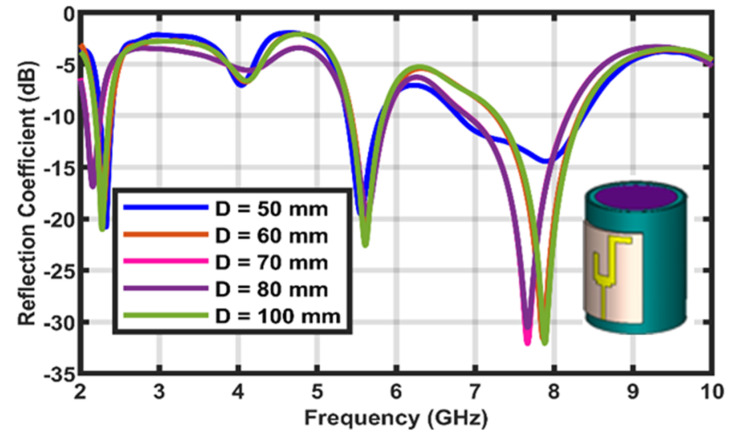
Effect of bending the antenna over different diameters.

To evaluate the antenna’s performance in terms of reflection coefficient, gain, and radiation pattern under bending conditions, we developed a three-layered cylindrical phantom using CST. The reflection coefficient of the antenna was analyzed under phantom loading conditions by varying the diameter of the inner cylinder from 50 to 100 mm, simulating changes in the size of the muscle layer. The thickness of the fat layer was kept constant at 2 mm, while the skin layer had a thickness of 1 mm. The tissue layers were assigned frequency-dependent dielectric properties, as specified in Table 3. Figure 18 illustrates the impact of this variation on the antenna’s reflection coefficient, demonstrating that the antenna exhibits a high level of resilience to various bending conditions while maintaining good impedance matching in the target resonance bands.

**Figure 19:**
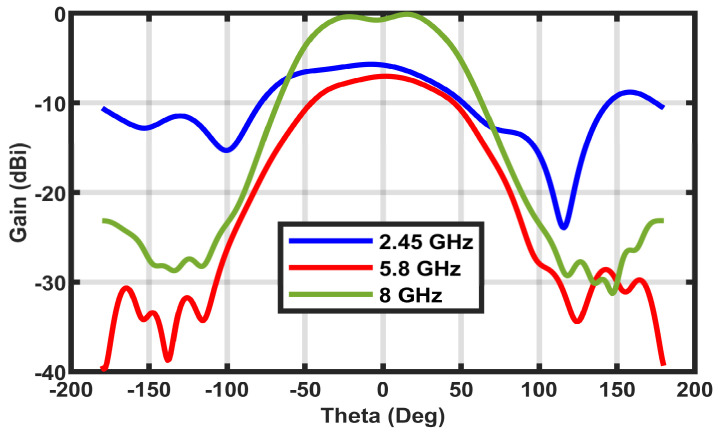
Variation in the gain of the antenna under phantom loading.

In another test, the antenna’s gain was measured at a distance of 3 mm from the skin layer, and the results were plotted as a function of angular distribution in the E-plane, as shown in Figure 19. From the figure, it is evident that the antenna’s gain decreased significantly under phantom loading, with the maximum gain approaching 0 dB. Notably, the same antenna achieved a maximum gain of 6.57 dB without phantom loading. Moreover, the −3 dB main lobe width increased for frequencies of 2.45 and 5.8 GHz compared to 8 GHz, resulting in less directive antenna performance in the former cases.

**Figure 20:**
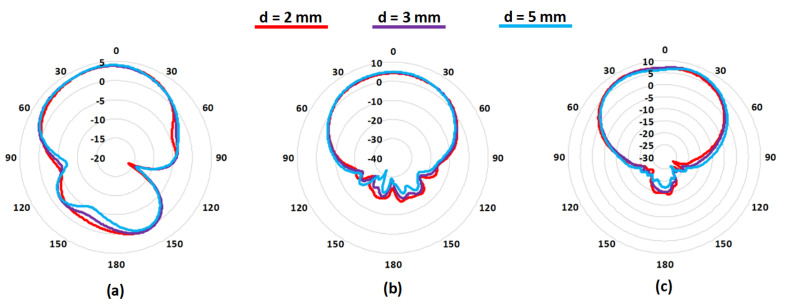
Simulated radiation pattern of the antenna in E-plane for various distances at frequencies of (**a**) 2.45 GHz, (**b**) 5.8 GHz, and (**c**) 8.0 GHz.

In body-worn communication, the distance between the antenna and the body can alter the antenna’s radiation-pattern characteristics. To explore this effect, we positioned the antenna at distances of 2, 3, and 5 mm from the skin layer and recorded the corresponding radiation patterns for the operating frequencies, as presented in Figure 20. As evident from the figure, the antenna maintains a high degree of stability in its radiation pattern for various distances. Additionally, the antenna exhibits a tendency towards symmetry in its radiation pattern as the distance between the antenna and the phantom increases.

### 5.4. Wet Test

**Figure 21:**
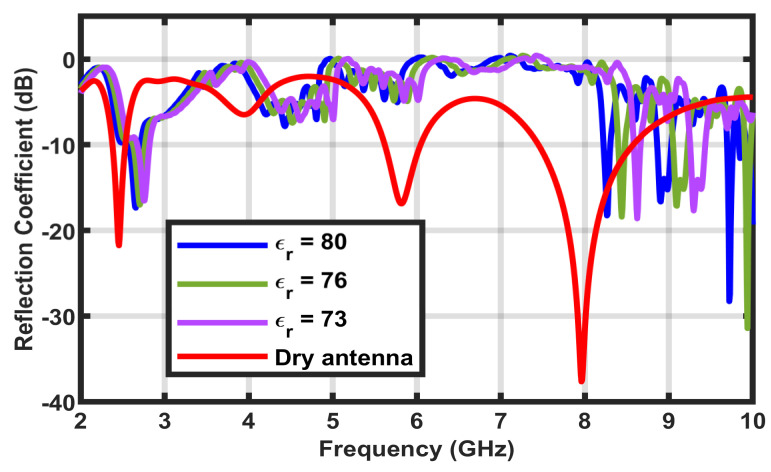
Reflection coefficient of the antenna under wet conditions.

The performance of the antenna can deteriorate in wet conditions, such as rainy or sweaty environments. To assess its resilience to wet conditions, we added a 1 mm thick layer above the antenna, fully covering its top layer, and filled it with water. We used three different permittivity values of water (80, 76, and 73) to simulate various levels of salt in the water. The results of this test are presented in Figure 21. As shown, the return loss profile of the antenna is significantly distorted due to the coupling of the antenna radiation with the high lossy medium of water. This interaction leads to a considerable amount of scattering of the antenna radiation.

### 5.5. Undercover Test

**Figure 22:**
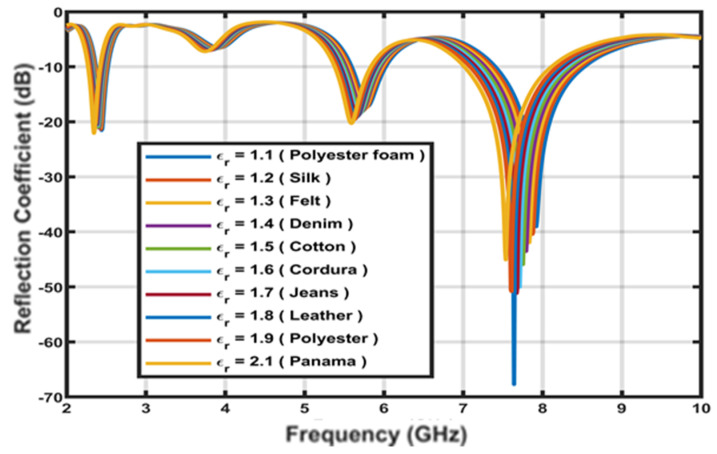
Reflection coefficient of the antenna for various undercover tests.

**Figure 23:**
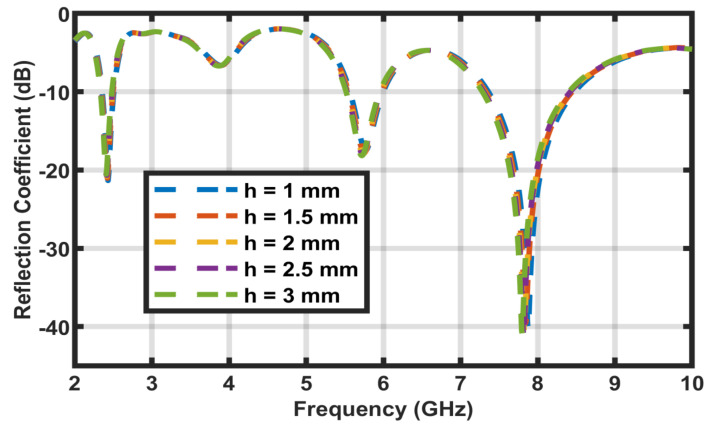
Reflection coefficient of the antenna for various thicknesses of the covering layer.

In practical body-worn communication scenarios, antennas are often placed on garments and exposed to free space. However, if the antenna is covered with body-worn fabrics made of cotton, silk, jeans, etc., its performance can be affected. To study this, a selection of ten body-worn fabrics was chosen, and the variation in return loss was measured with the antenna placed underneath a 1 mm thick layer. The results, shown in Figure 22, demonstrate that the antenna maintains stability in its resonance bands. Additionally, an analysis was performed to study the effect of increasing the thickness of the cover layer. Figure 23 displays the results, where “h” represents the thickness of the cover layer. It is evident that the antenna’s performance remains highly reliable for cover layer thicknesses up to 3 mm. Furthermore, the results suggest that the antenna can withstand thicker cover layers without any significant deterioration in its performance.

### 5.6. S_21_ Analysis

The transmission losses of the proposed wearable antenna can be investigated by evaluating the S_21_ parameter. This was accomplished first by configuring a pair of antennas in various positions in free space and then by mounting the antennas on the body.

**Figure 24:**
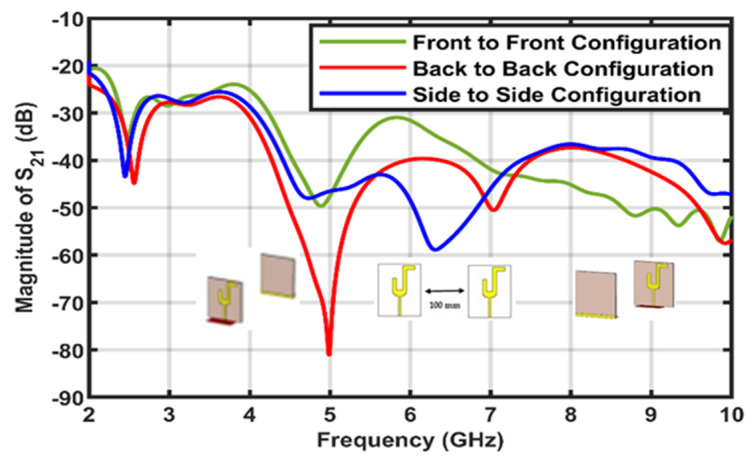
S_21_ analysis of the antenna for various configurations in free space.

**Figure 25:**
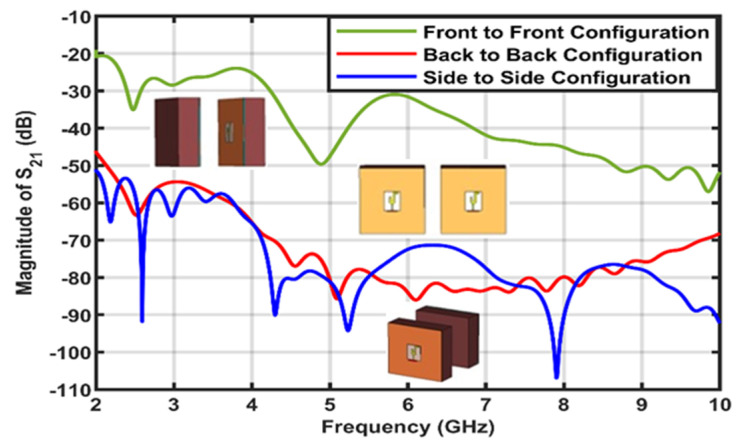
S_21_ analysis for the on-body antenna using various configurations.

These positions are defined as front to front, back to back, and side to side with 100 mm of separation. The result for the free-space case is presented in Figure 24. The transmission losses are around 25–85 dB. To evaluate on-body transmission losses, the pair of antennas are placed on a three-layered phantom model. The S_21_ parameter was then evaluated for three configurations. The results achieved are shown in Figure 25. The transmission losses for the on-body case vary between −20 and −110 dB. The low transmission-loss values for both cases indicate that the proposed antenna can establish a reliable communication link with nodes in free space or on the body.

### 5.7. SAR Simulation

The specific absorption rate (SAR) in W/Kg given by Equation (1) is used to quantify the antenna radiation absorbed by human tissue. A lower SAR value is preferable as per the guidelines set by the FCC.

(1) $$\mathrm{SAR}=\frac{{\sigma E}^{2}}{2\rho }$$

Here E is the E-field vector in (V/m), σ and ρ are the effective conductivity and density of the tissue layers given in (S/m) and (kg/m^3^), respectively [31,32,33]. A three-layered phantom model was developed in CST to assess SAR, consisting of a 50 mm-thick muscle layer, a 2 mm-thick fat layer, and a 1 mm-thick skin layer. These layers were assigned density values of 1020 Kg/m^3^, 909.4 Kg/m^3^, and 1060 Kg/m^3^ respectively. The antenna used in the model was positioned 3 mm away from the skin layer. SAR values were calculated for two different phantom shapes: a flat phantom with dimensions of 180 × 160 × 53 mm^3^ and a cylindrical phantom with a radius of 90 mm. The tissue layers were assigned frequency-dependent dielectric properties, and the second-order Debye’s model is used for dispersion fitting [34]. The input power for the SAR computation was set to 20 mW.

**Figure 26:**
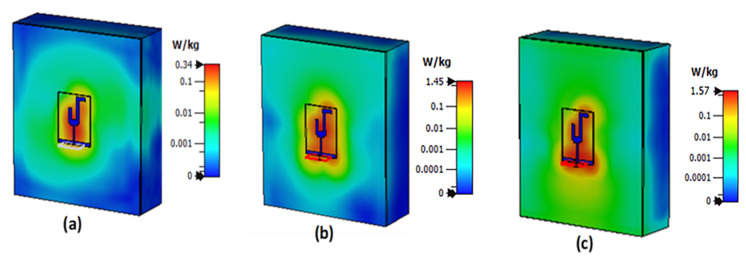
SAR_1g_ distribution for flat phantom (**a**) 2.45 GHz, (**b**) 5.8 GHz, and (**c**) 8.0 GHz.

**Figure 27:**
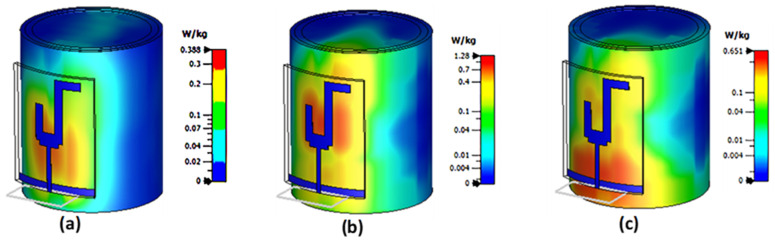
SAR_1g_ distribution for cylindrical phantom (**a**) 2.45 GHz, (**b**) 5.8 GHz, and (**c**) 8.0 GHz.

The simulation results obtained for SAR_1g_ are demonstrated in Figure 26 and Figure 27 for flat and cylindrical phantoms respectively. As depicted from these figures, the maximum value of SAR_1g_ is well below the FCC limit of 1.6 W/Kg.

## 6. Comparison with Related Works

There is rich literature available on the design of new antennas for wearable applications. However, a more suitable comparison for the proposed antenna would be with other antennas that use kapton polyimide as the substrate material. Table 4 presents this comparison, indicating that the proposed antenna has a higher gain compared to others. Additionally, the proposed antenna has three resonance bands that are not present in other antennas that use kapton polyimide as the substrate material.

## 7. Conclusions

The authors introduced a flexible multiband antenna capable of functioning in ISM, WLAN, and X-bands. The proposed antenna was designed on the flexible substrate of kapton polyimide material (permittivity value of 3.5) with a size of 52 mm × 40 mm (0.79 λ × 0.61 λ). The reflection coefficient of the antenna is significantly lower than −10 dB for all resonant frequency bands which reflects good impedance-matching characteristics. Appropriate gain values are achieved for the resonance bands with maximum efficiency of 83%. Various simulation tests were conducted to evaluate the antenna’s performance, and it was found that the SAR values were kept to a minimum. Overall, the antenna performed adequately for the target frequency bands.

## Figures and Tables

**Table 1:** Optimized dimension parameters and their values.

Parameter	Value (mm)	Parameter	Value (mm)	Parameter	Value (mm)
W	40	L	52	L_g_	3.2
W_F_	2.0	L_F_	20	L_L_	16
L_R_	24.5	L_E_	3.5	W_S_	6.0
W_L_	3.5	W_E_	11.5	C_1_	2.0
C_2_	2.0	W_R_	3.5		

**Table 2:** Bandwidth analysis of the proposed antenna.

Centre Frequencies	Frequency Range	S_11_ (dB)	Bandwidth (%)
2.45 GHz	2.37–2.54 GHz	−21.69	6.73
5.8 GHz	5.62–6.03 GHz	−16.86	7.02
8.0 GHz	7.45–8.56 GHz	−37.49	13.81

**Table 3:** Dielectric Properties of tissues at various frequencies.

Tissue	2.45 GHz		5.8 GHz		8.0 GHz	
	ε_r_	σ (S/m)	ε_r_	σ (S/m)	ε_r_	σ (S/m)
Skin	38	1.46	35.0	3.70	32.0	6.8
Fat	5.3	0.11	4.90	0.30	3.6	0.5
Muscle	52.7	1.77	48.48	4.96	44.5	8.9

**Table 4:** Comparison of the proposed antenna to other antennas having kapton polyimide substrate.

Ref.	Size (λ^2^)	Resonant Bands (GHz)	Gain (dBi)	Efficiency (%)	SAR_1g_/SAR_10g_ (W/Kg)
[35]	0.35 × 0.35	7–14	4	92	1.0
[36]	2.12 × 2.05	2.05–14	12.7	82	0.97
[37]	0.98 × 0.93	3.1–12	4.2	>90	0.16
[38]	0.28 × 0.25	1–4.3	NA	NA	NA
[39]	0.23 × 0.21	2.5/4.5	−5.34/−4.48	NA	0.21/0.57
[40]	1.14 × 0.76	2.45	4	NA	NA
[41]	3.05 × 0.67	3.2–13.0	9.98 with MIMO	NA	NA
[42]	0.51 × 0.27	2.45	7	NA	0.44
[43]	0.09 × 0.06	5–6	−0.35	12	1.99 for 10 g
[44]	0.14 × 0.06	0.4–0.8	1.6	NA	NA
[45]	0.58 × 0.64	2.48/3.9/5.1	3.6/5.7/4.1	62/75/63	0.07/0.05/0.02
**This work**	**0.79 × 0.61**	**2.45/5.8/8.0**	**0.36/4.82/6.57**	**65.8/83.8/76.4**	**0.34/1.45/1.57**

## Data Availability

Not applicable.
